# Ethyl 4-(2,4-difluoro­phen­yl)-6-methyl-1-phenyl-2-thioxo-1,2,3,4-tetra­hydro­pyrimidine-5-carboxyl­ate

**DOI:** 10.1107/S160053680901232X

**Published:** 2009-04-08

**Authors:** Hoong-Kun Fun, Samuel Robinson Jebas, M. Babu, B. Kalluraya

**Affiliations:** a X-ray Crystallography Unit, School of Physics, Universiti Sains Malaysia, 11800 USM, Penang, Malaysia; b Department of studies in Chemistry, Mangalore University, Mangalagangotri, Mangalore 574 199, India

## Abstract

The asymmetric unit of the title compound, C_20_H_18_F_2_N_2_O_2_S, contains four independent mol­ecules, two of which are paired into a dimer by way of two N—H⋯S hydrogen bonds. The other two independent mol­ecules are paired into two centrosymmetric dimers *via* pairs of inter­molecular N—H⋯S hydrogen bonds. In one mol­ecule, the carboxyl­ate O atoms, methyl­ene and methyl groups attached to the benzene ring are disordered between two positions in a 0.908 (3):0.092 (3) ratio. In two of the independent mol­ecules, the F and H atoms of the diflourophenyl ring are flip-flop disordered (*i.e.* by 180° about the C—C bond axis linking the ring to the rest of the molecule) in a 3:2 ratio. The crystal packing is stabilized by weak inter­molecular C—H⋯O hydrogen bonds.

## Related literature

For details of the synthesis, see: Kalluraya & Rai (2003 ▶); Kappe (1993 ▶); Steele *et al.* (1998 ▶). For the pharmaceutical applications of pyrimidine derivatives, see: Atwal (1990 ▶); Manjula *et al.* (2004 ▶); Sadanandam *et al.* (1992 ▶). For bond-length data, see: Allen *et al.* (1987 ▶). For ring puckering analysis, see: Cremer & Pople (1975 ▶). For stability of the temperature controller used for the data collection, see: Cosier & Glazer (1986 ▶).
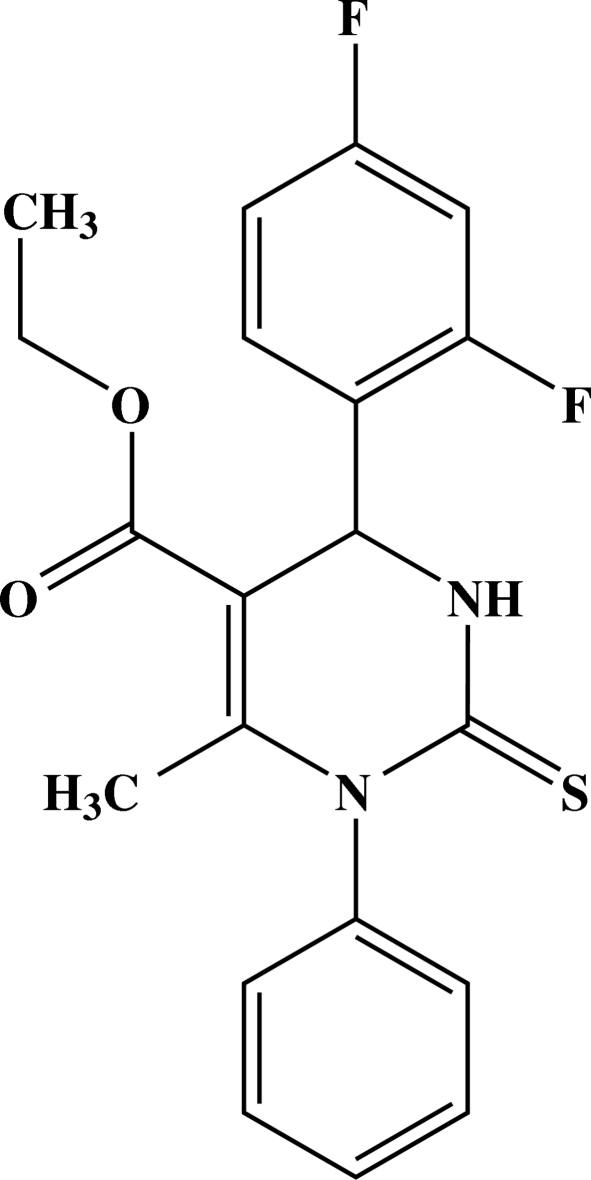


## Experimental

### 

#### Crystal data

C_20_H_18_F_2_N_2_O_2_S
*M* *_r_* = 388.42
Triclinic, 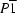

*a* = 11.3019 (2) Å
*b* = 13.9550 (2) Å
*c* = 24.3463 (4) Å
α = 98.807 (1)°
β = 93.776 (1)°
γ = 102.506 (1)°
*V* = 3684.98 (10) Å^3^
*Z* = 8
Mo *K*α radiation
μ = 0.21 mm^−1^
*T* = 100 K
0.57 × 0.36 × 0.14 mm

#### Data collection

Bruker SMART APEXII CCD area-detector diffractometer
Absorption correction: multi-scan (*SADABS*; Bruker, 2005 ▶) *T*_min_ = 0.888, *T*_max_ = 0.971
76910 measured reflections
21361 independent reflections
14585 reflections with *I* > 2σ(*I*)
*R*_int_ = 0.038

#### Refinement

*R*[*F* ^2^ > 2σ(*F* ^2^)] = 0.051
*wR*(*F* ^2^) = 0.133
*S* = 1.02
21361 reflections
1034 parameters
105 restraints
H atoms treated by a mixture of independent and constrained refinement
Δρ_max_ = 0.47 e Å^−3^
Δρ_min_ = −0.33 e Å^−3^

### 

Data collection: *APEX2* (Bruker, 2005 ▶); cell refinement: *SAINT* (Bruker, 2005 ▶); data reduction: *SAINT*; program(s) used to solve structure: *SHELXTL* (Sheldrick, 2008 ▶); program(s) used to refine structure: *SHELXTL*; molecular graphics: *SHELXTL*; software used to prepare material for publication: *SHELXTL* and *PLATON* (Spek, 2009 ▶).

## Supplementary Material

Crystal structure: contains datablocks global, I. DOI: 10.1107/S160053680901232X/cv2538sup1.cif

Structure factors: contains datablocks I. DOI: 10.1107/S160053680901232X/cv2538Isup2.hkl

Additional supplementary materials:  crystallographic information; 3D view; checkCIF report

## Figures and Tables

**Table 1:** Hydrogen-bond geometry (Å, °)

*D*—H⋯*A*	*D*—H	H⋯*A*	*D*⋯*A*	*D*—H⋯*A*
N2*A*—H1*NA*⋯S1*B*	0.86 (2)	2.63 (2)	3.4521 (15)	161.1 (19)
N2*B*—H1*NB*⋯S1*A*	0.87 (2)	2.624 (19)	3.4632 (15)	163.1 (18)
N2*C*—H1*NC*⋯S1*C*^i^	0.90 (2)	2.41 (2)	3.2686 (15)	160.0 (17)
N2*D*—H1*ND*⋯S1*D*^ii^	0.87 (2)	2.42 (2)	3.2537 (15)	159.7 (17)
C9*B*—H9*BA*⋯O2*C*^iii^	0.98	2.50	3.199 (2)	128
C16*C*—H16*A*⋯O2*A*^i^	0.93	2.46	3.143 (2)	130
C16*D*—H16*B*⋯O2*B*^iv^	0.93	2.47	3.146 (2)	130
C18*A*—H18*B*⋯O2*B*^v^	0.97	2.52	3.446 (3)	159
C18*B*—H18*C*⋯O2*A*^vi^	0.97	2.50	3.427 (2)	160

Symmetry codes: (i) 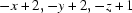; (ii) 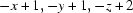; (iii) 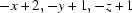; (iv) 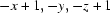; (v) 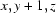; (vi) 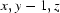.

**Fig. 1.**
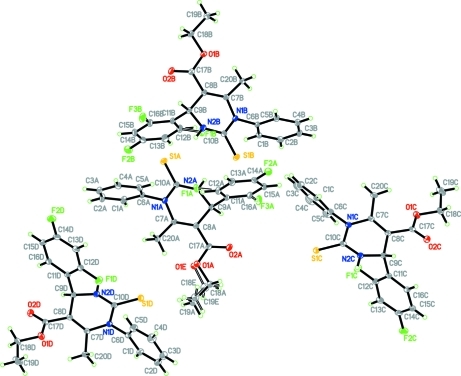
The content of asymmetric unit of the title compound showing the atom numbering scheme. Displacement ellipsoids are drawn at the 20% probability level.

## supplementary crystallographic information

### Comment

The subject of organic solid-state reactivity is a fascinating frontier area of research in the context of green chemistry. Michael addition followed by aldol condensation known as the Robinson's annulation is synthetically a very useful reaction for the construction of six membered cyclic compounds. (Kalluraya & Rai, 2003). 3,4-Dihydro-pyrimidinones are the compounds that have been drawn wide-spread attention, due to their pharmaceutical applications. (Atwal, 1990; Sadanandam *et al.*, 1992). The common synthetic routes to these compounds generally involve multi-step transformation that are essentially based on the Biginelli condensation methodology (Steele *et al.*, 1998). These pyrimidinones are also associated with activities like calcium channel blocking (Manjula *et al.*, 2004). In 1893, Biginelli reported the first synthesis of dihydropyrimidines by a simple one-pot condensation reaction of ethyl acetoacetate, benzaldehyde and urea. In the following decades the original *cyclo*-condensation reaction has been extended widely to include variations in all three components, allowing access to a large number of muti functionalized dihydropyrimidinone derivatives (Kappe, 1993). Due to the above interests, we report the synthesis of title compound (I) by means of Robinson's annulation employing microwave technique and its crystal structure.

The asymmetric unit of the title compound (Fig 1) contains four crystallographically independent molecules (A, B, C & D) with two molecules (C and D) exhibiting similar geometries and the other two molecules (A and B) displaying disorder. The bond lengths (Allen *et al.*, 1987) and angles are found to have normal values. Molecules A and B are linked together *via* intramolecular N—H···S hydrogen bonds into pseudo-centrosymmetric dimers whereas individual molecules C (and also D) are each linked *via* interamolecular N—H···S hydrogen bonds into centrosymmetric dimers. The pyrimidine ring exhibits non planarity with the maximum deviation of -0.1570 (19)Å for atom C9A, 0.1531 (19)Å for atom C9B, 0.1285 (16)Å for atom N2C and 0.1275 (16)Å for atom N2D. The pyrimidine rings of molecule A adopt the boat conformation with puckering parameters Q = 0.2403 (17) Å, θ = 75.0 (4)°, φ = 191.0 (4)°; molecule B and D adopting the twisted boat conformation with the puckering parameters Q = 0.2328 (17) Å, θ=107.7 (4)°, φ=14.1 (5)°; Q=0.2027 (17) Å, θ=111.1 (5) Å, φ=33.5 (5)° respectively; whereas molecule C adopting the screw boat conformation with the puckering parameters Q =0.1999 (17) Å, θ = 69.6 (5)°, φ = 217.6 (5)° (Cremer & Pople, 1975). The pyrimidine rings are almost orthogonal to the attached benzene rings in molecules A, B & D forming dihedral angles of 82.90 (8)° (N1A–N2A/C7A–C10A; C1A–C6A) and 87.22 (7)° (N1A–N2A/C7A–C10A; C11A–C16A); 88.76 (8)° (N1B–N2B/C7B–C10B; C1B–C6B) and 88.05 (7)° (N1B–N2B/C7B–C10B; C11B–C16B); and 83.53 (7)° N1D–N2D/C7D–C10D; C1D–C6D) and 81.68 (6)° (N1D–N2D/C7D–C10D; C11D–C16D) respectively. In molecule C, the pyrimidine ring forms dihedral angles of 68.65 (8)° (N1C–N2C/C7C–C10C; C1C–C6C) and 82.81 (8)° (N1C–N2C/C7C–C10C; C11C–C16C) with the attached benzene rings. In molecule A, the carboxylate oxygen, methylene and methyl groups (O1A/C18A/C19A) are disordered with occupancies of 0.908 (3):0.092 (3). The atoms F1A:H16C and H12A:F3A are flip-flop disordered with occupancies of 0.618 (3):0.382 (3). In molecule B, the atoms F1B:H16D and H12B:F3B are flip-flop disordered with occupancies of 0.382 (3):0.618 (3).

The crystal packing is stabilized by intermolecular C—H···O and N—H···S hydrogen bonding (Table 1).

### Experimental

A mixture of 2,4-difluoro benzaldehyde (0.01 mol), ethyl acetoacetate (0.015 mol), phenyl thiourea (0.01 mol) and conc. H2SO4 (2 drops) in absolute alcohol (10 ml) taken in a beaker (100 ml) was zapped inside a MW oven for a duration of three minutes (at 160 Watt). The reaction mixture was then allowed to stand at room temperature and the product formed was filtered, washed with ethanol followed by water and dried. Further purification was done by recrystallization from ethanol (yield 77%). Crystals suitable for X-ray analysis were obtained from ethanol by slow evaporation.

### Refinement

H atoms were positioned geometrically [C–H = 0.93–0.98Å (aromatic, methylene) or 0.96Å (methyl)] and refined using a riding model, with *U*_iso_(H) = 1.2*U*_eq_(aromatic C, methylene) and 1.5*U*_eq_(methyl C). A rotating–group model was used for the methyl groups. The N bound hydrogen atoms were located from the Fourier map and isotropically refined with the bond restraint N—H = 0.88 (2) Å. In molecule A the carboxylate oxygen, methylene and methyl groups (O1A/C18A/C19A) are disordered with occupancies of 0.902 (3):0.098 (3). The atoms F1A:H16C and H12A:F3A are flip-flop disordered with occupancies of 0.618 (3):0.382 (3). In molecule B, the atoms F1B:H16D and H12B:F3B are flip-flop disordered with the occupancies of 0.382 (3):0.618 (3). During the data collection, the temperature was controlled according to the literature procedure (Cosier & Glazer, 1986).

### Crystal data

**Table:**

C_20_H_18_F_2_N_2_O_2_S	*Z* = 8
*M**_r_* = 388.42	*F*(000) = 1616
Triclinic, *P*1	*D*_x_ = 1.400 Mg m^−^^3^
Hall symbol: -P 1	Mo *K*α radiation, λ = 0.71073 Å
*a* = 11.3019 (2) Å	Cell parameters from 9848 reflections
*b* = 13.9550 (2) Å	θ = 2.1–32.6°
*c* = 24.3463 (4) Å	µ = 0.21 mm^−^^1^
α = 98.807 (1)°	*T* = 100 K
β = 93.776 (1)°	Block, colourless
γ = 102.506 (1)°	0.57 × 0.36 × 0.14 mm
*V* = 3684.98 (10) Å^3^	

### Data collection

**Table:**

Bruker SMART APEXII CCD area-detector diffractometer	21361 independent reflections
Radiation source: fine-focus sealed tube	14585 reflections with *I* > 2σ(*I*)
graphite	*R*_int_ = 0.038
φ and ω scans	θ_max_ = 30.0°, θ_min_ = 0.9°
Absorption correction: multi-scan (*SADABS*; Bruker, 2005)	*h* = −15→15
*T*_min_ = 0.888, *T*_max_ = 0.971	*k* = −19→19
76910 measured reflections	*l* = −34→34

### Refinement

**Table:**

Refinement on *F*^2^	Primary atom site location: structure-invariant direct methods
Least-squares matrix: full	Secondary atom site location: difference Fourier map
*R*[*F*^2^ > 2σ(*F*^2^)] = 0.051	Hydrogen site location: inferred from neighbouring sites
*wR*(*F*^2^) = 0.133	H atoms treated by a mixture of independent and constrained refinement
*S* = 1.02	*w* = 1/[σ^2^(*F*_o_^2^) + (0.0548*P*)^2^ + 1.336*P*] where *P* = (*F*_o_^2^ + 2*F*_c_^2^)/3
21361 reflections	(Δ/σ)_max_ < 0.002
1034 parameters	Δρ_max_ = 0.47 e Å^−^^3^
105 restraints	Δρ_min_ = −0.33 e Å^−^^3^

### Special details

**Table:**

Experimental. The crystal was placed in the cold stream of an Oxford Cyrosystems Cobra open-flow nitrogen cryostat (Cosier & Glazer, 1986) operating at 100.0 (1) K.
Geometry. All e.s.d.'s (except the e.s.d. in the dihedral angle between two l.s. planes) are estimated using the full covariance matrix. The cell e.s.d.'s are taken into account individually in the estimation of e.s.d.'s in distances, angles and torsion angles; correlations between e.s.d.'s in cell parameters are only used when they are defined by crystal symmetry. An approximate (isotropic) treatment of cell e.s.d.'s is used for estimating e.s.d.'s involving l.s. planes.
Refinement. Refinement of *F*^2^ against ALL reflections. The weighted *R*-factor *wR* and goodness of fit *S* are based on *F*^2^, conventional *R*-factors *R* are based on *F*, with *F* set to zero for negative *F*^2^. The threshold expression of *F*^2^ > σ(*F*^2^) is used only for calculating *R*-factors(gt) *etc*. and is not relevant to the choice of reflections for refinement. *R*-factors based on *F*^2^ are statistically about twice as large as those based on *F*, and *R*- factors based on ALL data will be even larger.

### Fractional atomic coordinates and isotropic or equivalent isotropic displacement parameters (Å^2^)

**Table:**

	*x*	*y*	*z*	*U*_iso_*/*U*_eq_	Occ. (<1)
S1A	0.75838 (4)	0.19688 (3)	0.160405 (18)	0.02552 (10)	
O2A	0.88227 (12)	0.68085 (9)	0.25523 (5)	0.0299 (3)	
F2A	1.32384 (11)	0.55763 (8)	0.35317 (5)	0.0408 (3)	
N1A	0.82419 (13)	0.37625 (10)	0.12983 (5)	0.0210 (3)	
N2A	0.79954 (14)	0.37477 (10)	0.22300 (6)	0.0228 (3)	
C1A	0.73542 (17)	0.28783 (13)	0.03654 (7)	0.0297 (4)	
H1AA	0.6580	0.2880	0.0473	0.036*	
C2A	0.74994 (19)	0.25026 (15)	−0.01824 (8)	0.0372 (5)	
H2AA	0.6820	0.2259	−0.0444	0.045*	
C3A	0.8645 (2)	0.24892 (14)	−0.03401 (7)	0.0355 (5)	
H3AA	0.8738	0.2235	−0.0707	0.043*	
C4A	0.96586 (19)	0.28542 (15)	0.00479 (7)	0.0333 (4)	
H4AA	1.0432	0.2845	−0.0059	0.040*	
C5A	0.95199 (16)	0.32340 (13)	0.05967 (7)	0.0256 (4)	
H5AA	1.0197	0.3475	0.0859	0.031*	
C6A	0.83664 (15)	0.32491 (11)	0.07489 (6)	0.0199 (3)	
C7A	0.83666 (15)	0.48118 (11)	0.13756 (7)	0.0205 (3)	
C8A	0.84716 (15)	0.53280 (11)	0.18996 (6)	0.0203 (3)	
C9A	0.85118 (16)	0.48197 (12)	0.24026 (6)	0.0217 (3)	
H9AA	0.7990	0.5081	0.2665	0.026*	
C10A	0.79674 (15)	0.32235 (12)	0.17234 (7)	0.0209 (3)	
C11A	0.97837 (16)	0.50213 (11)	0.27052 (6)	0.0224 (3)	
C12A	1.07877 (17)	0.48994 (14)	0.24264 (7)	0.0293 (4)	
H12A	1.0670	0.4682	0.2042	0.035*	0.618 (3)
F1A	1.0585 (2)	0.4410 (2)	0.19055 (10)	0.0277 (8)	0.382 (3)
C13A	1.19565 (18)	0.50857 (14)	0.26922 (8)	0.0325 (4)	
H13A	1.2614	0.5003	0.2494	0.039*	
C14A	1.21032 (17)	0.53975 (12)	0.32601 (8)	0.0297 (4)	
C15A	1.11583 (19)	0.55307 (13)	0.35638 (7)	0.0312 (4)	
H15A	1.1284	0.5741	0.3949	0.037*	
C16A	1.00078 (18)	0.53433 (12)	0.32796 (7)	0.0275 (4)	
H16C	0.9359	0.5436	0.3481	0.033*	0.382 (3)
F3A	0.90942 (17)	0.54105 (14)	0.35765 (7)	0.0382 (6)	0.618 (3)
C17A	0.86280 (16)	0.64179 (12)	0.20659 (7)	0.0245 (3)	
O1A	0.86007 (18)	0.69379 (10)	0.16543 (5)	0.0275 (4)	0.908 (3)
C18A	0.8829 (2)	0.80187 (14)	0.18231 (8)	0.0290 (5)	0.908 (3)
H18A	0.9647	0.8281	0.2009	0.035*	0.908 (3)
H18B	0.8252	0.8190	0.2077	0.035*	0.908 (3)
C19A	0.8681 (2)	0.84402 (16)	0.12969 (9)	0.0333 (5)	0.908 (3)
H19A	0.8820	0.9152	0.1388	0.050*	0.908 (3)
H19B	0.7870	0.8171	0.1117	0.050*	0.908 (3)
H19C	0.9259	0.8266	0.1050	0.050*	0.908 (3)
O1E	0.7873 (18)	0.6727 (9)	0.1611 (6)	0.0275 (4)	0.092 (3)
C18E	0.797 (2)	0.7800 (11)	0.1680 (9)	0.030 (4)	0.092 (3)
H18I	0.7242	0.7932	0.1502	0.036*	0.092 (3)
H18J	0.8045	0.8081	0.2075	0.036*	0.092 (3)
C19E	0.905 (2)	0.8257 (16)	0.1425 (10)	0.0333 (5)	0.092 (3)
H19M	0.9065	0.8948	0.1424	0.050*	0.092 (3)
H19N	0.9015	0.7920	0.1047	0.050*	0.092 (3)
H19O	0.9773	0.8202	0.1635	0.050*	0.092 (3)
C20A	0.83525 (18)	0.52424 (13)	0.08446 (7)	0.0277 (4)	
H20A	0.8348	0.5936	0.0932	0.041*	
H20B	0.7636	0.4896	0.0601	0.041*	
H20C	0.9064	0.5170	0.0663	0.041*	
S1B	0.75338 (4)	0.28831 (3)	0.347157 (17)	0.02507 (10)	
O1B	0.65299 (12)	−0.20878 (8)	0.34331 (5)	0.0276 (3)	
O2B	0.62896 (12)	−0.19589 (9)	0.25299 (5)	0.0313 (3)	
F2B	0.21354 (12)	−0.05966 (9)	0.14024 (5)	0.0551 (4)	
N1B	0.68304 (13)	0.10938 (10)	0.37749 (5)	0.0201 (3)	
N2B	0.72065 (14)	0.11022 (10)	0.28549 (6)	0.0234 (3)	
C1B	0.55091 (15)	0.17504 (12)	0.44143 (7)	0.0231 (3)	
H1BA	0.4869	0.1542	0.4131	0.028*	
C2B	0.53163 (17)	0.21904 (13)	0.49421 (7)	0.0278 (4)	
H2BA	0.4545	0.2279	0.5013	0.033*	
C3B	0.62774 (19)	0.24983 (13)	0.53636 (7)	0.0308 (4)	
H3BA	0.6150	0.2793	0.5717	0.037*	
C4B	0.74210 (18)	0.23656 (13)	0.52558 (7)	0.0304 (4)	
H4BA	0.8062	0.2569	0.5539	0.037*	
C5B	0.76198 (16)	0.19320 (12)	0.47299 (7)	0.0252 (4)	
H5BA	0.8392	0.1848	0.4658	0.030*	
C6B	0.66595 (15)	0.16241 (11)	0.43125 (6)	0.0189 (3)	
C7B	0.66750 (14)	0.00404 (11)	0.37000 (7)	0.0200 (3)	
C8B	0.66290 (15)	−0.04792 (11)	0.31810 (6)	0.0204 (3)	
C9B	0.66849 (16)	0.00325 (12)	0.26761 (7)	0.0224 (3)	
H9BA	0.7238	−0.0236	0.2434	0.027*	
C10B	0.71625 (15)	0.16289 (12)	0.33534 (7)	0.0205 (3)	
C11B	0.54581 (16)	−0.01458 (12)	0.23358 (7)	0.0243 (4)	
C12B	0.44191 (18)	−0.00158 (14)	0.25786 (8)	0.0331 (4)	
H12B	0.4481	0.0186	0.2964	0.040*	0.618 (3)
F1B	0.4577 (2)	0.0489 (2)	0.30888 (10)	0.0306 (8)	0.382 (3)
C13B	0.32909 (19)	−0.01707 (15)	0.22775 (9)	0.0391 (5)	
H13B	0.2606	−0.0085	0.2453	0.047*	
C14B	0.3225 (2)	−0.04555 (13)	0.17092 (8)	0.0383 (5)	
C15B	0.4209 (2)	−0.05954 (14)	0.14403 (8)	0.0410 (5)	
H15B	0.4140	−0.0788	0.1054	0.049*	
C16B	0.53175 (19)	−0.04414 (13)	0.17595 (7)	0.0321 (4)	
H16D	0.5994	−0.0540	0.1580	0.039*	0.382 (3)
F3B	0.62696 (19)	−0.05051 (14)	0.14996 (7)	0.0416 (6)	0.618 (3)
C17B	0.64673 (15)	−0.15711 (12)	0.30179 (7)	0.0229 (3)	
C18B	0.63071 (18)	−0.31692 (12)	0.32629 (7)	0.0300 (4)	
H18C	0.6890	−0.3335	0.3010	0.036*	
H18D	0.5493	−0.3431	0.3073	0.036*	
C19B	0.64424 (19)	−0.36055 (14)	0.37831 (8)	0.0358 (4)	
H19D	0.6324	−0.4315	0.3685	0.054*	
H19E	0.5846	−0.3453	0.4025	0.054*	
H19F	0.7244	−0.3328	0.3972	0.054*	
C20B	0.65746 (17)	−0.03903 (12)	0.42309 (7)	0.0254 (4)	
H20D	0.6340	−0.1104	0.4140	0.038*	
H20E	0.5972	−0.0148	0.4437	0.038*	
H20F	0.7348	−0.0193	0.4454	0.038*	
S1C	0.92583 (4)	0.83676 (3)	0.468491 (17)	0.02691 (10)	
O1C	0.92847 (12)	0.84445 (10)	0.74524 (5)	0.0323 (3)	
O2C	1.04966 (12)	0.99326 (9)	0.74164 (5)	0.0315 (3)	
F1C	0.74070 (9)	0.91926 (7)	0.58298 (4)	0.0306 (2)	
F2C	0.65175 (11)	1.21670 (9)	0.66815 (5)	0.0391 (3)	
N1C	0.91419 (13)	0.77793 (10)	0.56803 (6)	0.0214 (3)	
N2C	0.99707 (13)	0.94456 (10)	0.56861 (6)	0.0211 (3)	
C1C	0.9145 (2)	0.62030 (13)	0.50690 (8)	0.0337 (4)	
H1CA	0.9970	0.6430	0.5038	0.040*	
C2C	0.8524 (3)	0.52555 (15)	0.48020 (9)	0.0495 (6)	
H2CA	0.8934	0.4857	0.4581	0.059*	
C3C	0.7326 (3)	0.49036 (17)	0.48598 (11)	0.0642 (8)	
H3CA	0.6924	0.4266	0.4684	0.077*	
C4C	0.6715 (3)	0.5496 (2)	0.51788 (13)	0.0709 (9)	
H4CA	0.5900	0.5252	0.5224	0.085*	
C5C	0.73056 (19)	0.64611 (17)	0.54357 (10)	0.0471 (6)	
H5CA	0.6883	0.6869	0.5641	0.057*	
C6C	0.85196 (16)	0.68006 (12)	0.53807 (7)	0.0257 (4)	
C7C	0.93441 (15)	0.79305 (12)	0.62721 (7)	0.0212 (3)	
C8C	0.96774 (15)	0.88628 (12)	0.65661 (7)	0.0214 (3)	
C9C	0.99104 (15)	0.97668 (12)	0.62817 (6)	0.0199 (3)	
H9CA	1.0715	1.0177	0.6437	0.024*	
C10C	0.94492 (15)	0.85531 (12)	0.53858 (7)	0.0207 (3)	
C11C	0.89957 (15)	1.04126 (12)	0.63833 (6)	0.0202 (3)	
C12C	0.77895 (15)	1.01087 (12)	0.61542 (7)	0.0226 (3)	
C13C	0.69455 (16)	1.06869 (13)	0.62349 (7)	0.0271 (4)	
H13C	0.6150	1.0472	0.6065	0.033*	
C14C	0.73425 (17)	1.16003 (13)	0.65805 (7)	0.0276 (4)	
C15C	0.85183 (17)	1.19531 (13)	0.68213 (7)	0.0274 (4)	
H15C	0.8759	1.2575	0.7049	0.033*	
C17C	0.98855 (16)	0.91343 (13)	0.71814 (7)	0.0244 (3)	
C18C	0.9448 (2)	0.86862 (15)	0.80605 (7)	0.0372 (5)	
H18E	0.9032	0.9204	0.8191	0.045*	
H18F	1.0306	0.8917	0.8194	0.045*	
C19C	0.8910 (2)	0.77396 (18)	0.82669 (9)	0.0488 (6)	
H19G	0.8957	0.7868	0.8667	0.073*	
H19H	0.9358	0.7246	0.8150	0.073*	
H19I	0.8074	0.7501	0.8115	0.073*	
C20C	0.92231 (17)	0.69861 (12)	0.65126 (7)	0.0276 (4)	
H20G	0.9666	0.7132	0.6877	0.041*	
H20H	0.9547	0.6515	0.6273	0.041*	
H20I	0.8379	0.6710	0.6541	0.041*	
S1D	0.56431 (4)	0.66307 (3)	1.034717 (17)	0.02846 (10)	
O1D	0.56232 (13)	0.66675 (10)	0.75837 (5)	0.0343 (3)	
O2D	0.43475 (12)	0.52084 (10)	0.76128 (5)	0.0335 (3)	
F2D	0.83022 (11)	0.28243 (9)	0.83084 (5)	0.0396 (3)	
F1D	0.75066 (9)	0.58129 (8)	0.91963 (4)	0.0316 (2)	
N1D	0.58169 (13)	0.72586 (10)	0.93662 (5)	0.0212 (3)	
N2D	0.49398 (13)	0.55931 (10)	0.93340 (6)	0.0215 (3)	
C1D	0.59595 (18)	0.88982 (13)	0.99457 (7)	0.0305 (4)	
H1DA	0.5114	0.8781	0.9920	0.037*	
C2D	0.6675 (2)	0.97884 (14)	1.02477 (8)	0.0374 (5)	
H2DA	0.6305	1.0269	1.0423	0.045*	
C3D	0.7924 (2)	0.99603 (15)	1.02882 (9)	0.0437 (5)	
H3DA	0.8396	1.0562	1.0482	0.052*	
C4D	0.8475 (2)	0.92390 (16)	1.00414 (9)	0.0436 (5)	
H4DA	0.9319	0.9344	1.0081	0.052*	
C5D	0.77699 (17)	0.83532 (14)	0.97322 (8)	0.0327 (4)	
H5DA	0.8141	0.7872	0.9558	0.039*	
C6D	0.65208 (15)	0.81957 (12)	0.96858 (7)	0.0223 (3)	
C7D	0.56196 (15)	0.71469 (12)	0.87761 (7)	0.0215 (3)	
C8D	0.52512 (15)	0.62249 (12)	0.84687 (7)	0.0219 (3)	
C9D	0.50040 (15)	0.53038 (11)	0.87357 (6)	0.0204 (3)	
H9DA	0.4196	0.4907	0.8575	0.024*	
C10D	0.54763 (15)	0.64690 (12)	0.96469 (7)	0.0211 (3)	
C11D	0.59026 (15)	0.46450 (11)	0.86199 (6)	0.0203 (3)	
C12D	0.71052 (15)	0.49119 (12)	0.88551 (7)	0.0235 (3)	
C13D	0.79177 (17)	0.43117 (13)	0.87703 (7)	0.0282 (4)	
H13D	0.8709	0.4502	0.8946	0.034*	
C14D	0.75037 (17)	0.34137 (13)	0.84127 (7)	0.0287 (4)	
C15D	0.63312 (17)	0.30988 (13)	0.81591 (7)	0.0274 (4)	
H15D	0.6078	0.2489	0.7921	0.033*	
C16C	0.93428 (16)	1.13519 (12)	0.67144 (7)	0.0232 (3)	
H16A	1.0148	1.1586	0.6869	0.028*	
C17D	0.50025 (16)	0.59825 (13)	0.78517 (7)	0.0257 (4)	
C18D	0.5410 (2)	0.64679 (16)	0.69772 (7)	0.0406 (5)	
H18G	0.4544	0.6272	0.6857	0.049*	
H18H	0.5787	0.5938	0.6824	0.049*	
C19D	0.5972 (3)	0.7420 (2)	0.67881 (9)	0.0572 (7)	
H19J	0.5875	0.7325	0.6387	0.086*	
H19K	0.6824	0.7613	0.6919	0.086*	
H19L	0.5578	0.7933	0.6937	0.086*	
C20D	0.57818 (18)	0.81196 (12)	0.85654 (7)	0.0281 (4)	
H20J	0.5495	0.7996	0.8175	0.042*	
H20K	0.6629	0.8451	0.8617	0.042*	
H20L	0.5325	0.8533	0.8770	0.042*	
C16D	0.55333 (16)	0.37216 (12)	0.82703 (7)	0.0235 (3)	
H16B	0.4733	0.3516	0.8107	0.028*	
H1NA	0.777 (2)	0.3407 (16)	0.2484 (9)	0.042 (6)*	
H1NB	0.7418 (18)	0.1428 (15)	0.2587 (8)	0.031 (5)*	
H1NC	1.0197 (19)	0.9954 (16)	0.5500 (8)	0.037 (6)*	
H1ND	0.4711 (18)	0.5074 (15)	0.9495 (8)	0.031 (5)*	

### Atomic displacement parameters (Å^2^)

**Table:**

	*U*^11^	*U*^22^	*U*^33^	*U*^12^	*U*^13^	*U*^23^
S1A	0.0325 (2)	0.01602 (19)	0.0260 (2)	0.00129 (17)	0.00659 (17)	0.00182 (15)
O2A	0.0411 (8)	0.0207 (6)	0.0250 (6)	0.0053 (5)	0.0032 (5)	−0.0019 (5)
F2A	0.0426 (7)	0.0315 (6)	0.0428 (7)	0.0079 (5)	−0.0182 (5)	0.0001 (5)
N1A	0.0281 (8)	0.0162 (6)	0.0171 (6)	0.0028 (6)	0.0038 (5)	0.0005 (5)
N2A	0.0293 (8)	0.0172 (7)	0.0208 (7)	0.0013 (6)	0.0070 (6)	0.0033 (5)
C1A	0.0258 (9)	0.0293 (9)	0.0291 (9)	0.0025 (7)	−0.0010 (7)	−0.0037 (7)
C2A	0.0433 (12)	0.0343 (10)	0.0258 (9)	0.0030 (9)	−0.0084 (8)	−0.0064 (8)
C3A	0.0594 (14)	0.0290 (10)	0.0195 (8)	0.0155 (9)	0.0059 (8)	0.0003 (7)
C4A	0.0418 (11)	0.0415 (11)	0.0245 (9)	0.0209 (9)	0.0122 (8)	0.0100 (8)
C5A	0.0262 (9)	0.0303 (9)	0.0213 (8)	0.0079 (7)	0.0011 (7)	0.0061 (7)
C6A	0.0261 (9)	0.0153 (7)	0.0166 (7)	0.0032 (6)	0.0007 (6)	−0.0001 (6)
C7A	0.0226 (8)	0.0154 (7)	0.0226 (8)	0.0032 (6)	0.0024 (6)	0.0022 (6)
C8A	0.0229 (8)	0.0172 (7)	0.0198 (7)	0.0028 (6)	0.0030 (6)	0.0021 (6)
C9A	0.0286 (9)	0.0169 (7)	0.0186 (7)	0.0034 (7)	0.0071 (6)	0.0007 (6)
C10A	0.0202 (8)	0.0186 (8)	0.0228 (8)	0.0021 (6)	0.0038 (6)	0.0022 (6)
C11A	0.0316 (9)	0.0146 (7)	0.0194 (8)	0.0018 (7)	0.0025 (7)	0.0029 (6)
C12A	0.0321 (10)	0.0310 (9)	0.0218 (8)	0.0058 (8)	0.0018 (7)	−0.0023 (7)
F1A	0.0273 (15)	0.0395 (17)	0.0143 (12)	0.0068 (12)	0.0003 (10)	0.0008 (11)
C13A	0.0298 (10)	0.0342 (10)	0.0322 (10)	0.0091 (8)	−0.0004 (8)	0.0012 (8)
C14A	0.0361 (11)	0.0184 (8)	0.0311 (9)	0.0024 (7)	−0.0100 (8)	0.0045 (7)
C15A	0.0496 (12)	0.0199 (8)	0.0199 (8)	0.0018 (8)	−0.0051 (8)	0.0030 (6)
C16A	0.0405 (11)	0.0196 (8)	0.0197 (8)	0.0017 (7)	0.0049 (7)	0.0023 (6)
F3A	0.0459 (12)	0.0459 (12)	0.0211 (9)	0.0097 (9)	0.0133 (8)	−0.0025 (7)
C17A	0.0297 (9)	0.0196 (8)	0.0244 (8)	0.0059 (7)	0.0070 (7)	0.0023 (6)
O1A	0.0434 (11)	0.0168 (6)	0.0220 (6)	0.0071 (7)	0.0036 (7)	0.0018 (5)
C18A	0.0447 (14)	0.0155 (9)	0.0261 (10)	0.0073 (9)	0.0068 (9)	−0.0008 (7)
C19A	0.0486 (15)	0.0220 (10)	0.0293 (11)	0.0073 (9)	0.0021 (10)	0.0059 (8)
O1E	0.0434 (11)	0.0168 (6)	0.0220 (6)	0.0071 (7)	0.0036 (7)	0.0018 (5)
C18E	0.048 (10)	0.015 (7)	0.032 (9)	0.018 (7)	0.011 (8)	0.004 (7)
C19E	0.0486 (15)	0.0220 (10)	0.0293 (11)	0.0073 (9)	0.0021 (10)	0.0059 (8)
C20A	0.0416 (11)	0.0218 (8)	0.0192 (8)	0.0079 (8)	0.0005 (7)	0.0026 (6)
S1B	0.0324 (2)	0.01644 (19)	0.0247 (2)	0.00171 (17)	0.00731 (17)	0.00205 (15)
O1B	0.0398 (7)	0.0176 (6)	0.0249 (6)	0.0085 (5)	0.0001 (5)	0.0003 (5)
O2B	0.0456 (8)	0.0214 (6)	0.0236 (6)	0.0054 (6)	0.0024 (5)	−0.0029 (5)
F2B	0.0644 (9)	0.0343 (7)	0.0563 (8)	0.0086 (6)	−0.0389 (7)	−0.0011 (6)
N1B	0.0250 (7)	0.0156 (6)	0.0179 (6)	0.0026 (5)	0.0032 (5)	0.0003 (5)
N2B	0.0291 (8)	0.0182 (7)	0.0211 (7)	0.0007 (6)	0.0075 (6)	0.0023 (5)
C1B	0.0255 (9)	0.0233 (8)	0.0210 (8)	0.0061 (7)	0.0012 (6)	0.0054 (6)
C2B	0.0327 (10)	0.0291 (9)	0.0267 (9)	0.0134 (8)	0.0103 (7)	0.0081 (7)
C3B	0.0513 (12)	0.0234 (9)	0.0179 (8)	0.0098 (8)	0.0063 (8)	0.0016 (6)
C4B	0.0392 (11)	0.0248 (9)	0.0222 (8)	0.0026 (8)	−0.0066 (7)	−0.0011 (7)
C5B	0.0237 (9)	0.0235 (8)	0.0259 (8)	0.0040 (7)	−0.0013 (7)	0.0004 (7)
C6B	0.0243 (8)	0.0147 (7)	0.0168 (7)	0.0034 (6)	0.0026 (6)	0.0013 (6)
C7B	0.0198 (8)	0.0176 (7)	0.0217 (8)	0.0031 (6)	0.0013 (6)	0.0024 (6)
C8B	0.0205 (8)	0.0179 (7)	0.0212 (8)	0.0025 (6)	0.0022 (6)	0.0014 (6)
C9B	0.0271 (9)	0.0170 (7)	0.0209 (8)	0.0015 (7)	0.0061 (7)	−0.0003 (6)
C10B	0.0206 (8)	0.0175 (7)	0.0218 (8)	0.0020 (6)	0.0035 (6)	0.0015 (6)
C11B	0.0353 (10)	0.0166 (8)	0.0186 (8)	0.0013 (7)	0.0013 (7)	0.0027 (6)
C12B	0.0360 (11)	0.0347 (10)	0.0252 (9)	0.0101 (8)	−0.0047 (8)	−0.0045 (7)
F1B	0.0294 (16)	0.0420 (17)	0.0199 (14)	0.0105 (13)	0.0006 (11)	0.0006 (11)
C13B	0.0363 (11)	0.0363 (11)	0.0413 (11)	0.0104 (9)	−0.0094 (9)	−0.0010 (9)
C14B	0.0507 (13)	0.0195 (9)	0.0377 (11)	0.0027 (8)	−0.0219 (10)	0.0025 (8)
C15B	0.0713 (16)	0.0230 (9)	0.0209 (9)	0.0001 (10)	−0.0124 (9)	0.0017 (7)
C16B	0.0499 (12)	0.0222 (9)	0.0199 (8)	−0.0006 (8)	0.0039 (8)	0.0028 (7)
F3B	0.0603 (14)	0.0411 (11)	0.0218 (9)	0.0099 (10)	0.0153 (8)	−0.0017 (8)
C17B	0.0231 (9)	0.0196 (8)	0.0251 (8)	0.0047 (7)	0.0035 (7)	0.0009 (6)
C18B	0.0422 (11)	0.0164 (8)	0.0301 (9)	0.0060 (8)	0.0069 (8)	−0.0003 (7)
C19B	0.0468 (12)	0.0237 (9)	0.0353 (10)	0.0075 (8)	−0.0025 (9)	0.0042 (8)
C20B	0.0360 (10)	0.0209 (8)	0.0203 (8)	0.0079 (7)	0.0023 (7)	0.0045 (6)
S1C	0.0386 (3)	0.0189 (2)	0.0221 (2)	0.00405 (18)	−0.00055 (18)	0.00549 (16)
O1C	0.0438 (8)	0.0327 (7)	0.0214 (6)	0.0080 (6)	0.0025 (5)	0.0092 (5)
O2C	0.0387 (8)	0.0268 (7)	0.0277 (6)	0.0094 (6)	−0.0021 (6)	0.0008 (5)
F1C	0.0208 (5)	0.0248 (5)	0.0417 (6)	0.0013 (4)	−0.0012 (4)	−0.0007 (4)
F2C	0.0432 (7)	0.0407 (7)	0.0440 (7)	0.0280 (6)	0.0102 (5)	0.0113 (5)
N1C	0.0242 (7)	0.0172 (6)	0.0225 (7)	0.0031 (6)	0.0006 (6)	0.0050 (5)
N2C	0.0233 (7)	0.0183 (7)	0.0222 (7)	0.0035 (6)	0.0047 (6)	0.0061 (5)
C1C	0.0488 (12)	0.0181 (8)	0.0327 (10)	0.0033 (8)	0.0033 (9)	0.0059 (7)
C2C	0.090 (2)	0.0185 (9)	0.0338 (11)	0.0032 (11)	−0.0091 (11)	0.0056 (8)
C3C	0.091 (2)	0.0265 (11)	0.0571 (15)	−0.0170 (13)	−0.0361 (15)	0.0122 (11)
C4C	0.0472 (16)	0.0550 (17)	0.092 (2)	−0.0252 (13)	−0.0304 (15)	0.0246 (16)
C5C	0.0293 (11)	0.0454 (13)	0.0604 (14)	−0.0040 (9)	−0.0074 (10)	0.0130 (11)
C6C	0.0297 (10)	0.0177 (8)	0.0268 (8)	−0.0016 (7)	−0.0043 (7)	0.0082 (6)
C7C	0.0200 (8)	0.0219 (8)	0.0231 (8)	0.0049 (6)	0.0016 (6)	0.0089 (6)
C8C	0.0211 (8)	0.0209 (8)	0.0238 (8)	0.0062 (6)	0.0013 (6)	0.0069 (6)
C9C	0.0192 (8)	0.0184 (7)	0.0217 (8)	0.0033 (6)	0.0012 (6)	0.0040 (6)
C10C	0.0188 (8)	0.0198 (8)	0.0246 (8)	0.0056 (6)	0.0025 (6)	0.0059 (6)
C11C	0.0219 (8)	0.0190 (7)	0.0212 (8)	0.0054 (6)	0.0035 (6)	0.0070 (6)
C12C	0.0231 (9)	0.0202 (8)	0.0245 (8)	0.0034 (7)	0.0029 (7)	0.0062 (6)
C13C	0.0237 (9)	0.0308 (9)	0.0306 (9)	0.0093 (7)	0.0048 (7)	0.0119 (7)
C14C	0.0332 (10)	0.0292 (9)	0.0292 (9)	0.0181 (8)	0.0110 (7)	0.0135 (7)
C15C	0.0373 (10)	0.0230 (8)	0.0253 (8)	0.0117 (8)	0.0054 (7)	0.0069 (7)
C17C	0.0261 (9)	0.0243 (8)	0.0256 (8)	0.0114 (7)	−0.0008 (7)	0.0067 (7)
C18C	0.0513 (13)	0.0451 (12)	0.0225 (9)	0.0248 (10)	0.0035 (8)	0.0084 (8)
C19C	0.0567 (15)	0.0683 (16)	0.0344 (11)	0.0258 (13)	0.0153 (10)	0.0282 (11)
C20C	0.0339 (10)	0.0208 (8)	0.0305 (9)	0.0072 (7)	0.0018 (7)	0.0114 (7)
S1D	0.0427 (3)	0.0190 (2)	0.0208 (2)	0.00048 (18)	0.00164 (18)	0.00446 (15)
O1D	0.0487 (9)	0.0343 (7)	0.0211 (6)	0.0100 (6)	0.0015 (6)	0.0081 (5)
O2D	0.0386 (8)	0.0307 (7)	0.0279 (7)	0.0092 (6)	−0.0067 (6)	−0.0021 (5)
F2D	0.0449 (7)	0.0402 (7)	0.0433 (7)	0.0274 (6)	0.0087 (5)	0.0094 (5)
F1D	0.0228 (5)	0.0261 (5)	0.0397 (6)	0.0011 (4)	−0.0038 (4)	−0.0036 (4)
N1D	0.0254 (8)	0.0168 (6)	0.0198 (6)	0.0014 (6)	0.0009 (5)	0.0038 (5)
N2D	0.0241 (8)	0.0167 (7)	0.0222 (7)	0.0007 (6)	0.0033 (6)	0.0039 (5)
C1D	0.0350 (10)	0.0241 (9)	0.0319 (9)	0.0067 (8)	0.0026 (8)	0.0033 (7)
C2D	0.0558 (14)	0.0211 (9)	0.0338 (10)	0.0081 (9)	0.0018 (9)	0.0020 (7)
C3D	0.0560 (14)	0.0212 (9)	0.0426 (12)	−0.0113 (9)	−0.0039 (10)	0.0032 (8)
C4D	0.0360 (12)	0.0374 (12)	0.0497 (13)	−0.0098 (9)	0.0012 (10)	0.0107 (10)
C5D	0.0312 (10)	0.0271 (9)	0.0371 (10)	−0.0005 (8)	0.0064 (8)	0.0064 (8)
C6D	0.0265 (9)	0.0165 (7)	0.0217 (8)	−0.0001 (6)	0.0009 (7)	0.0050 (6)
C7D	0.0219 (8)	0.0218 (8)	0.0218 (8)	0.0053 (7)	0.0022 (6)	0.0066 (6)
C8D	0.0225 (8)	0.0216 (8)	0.0223 (8)	0.0066 (7)	0.0001 (6)	0.0048 (6)
C9D	0.0197 (8)	0.0176 (7)	0.0223 (8)	0.0017 (6)	0.0005 (6)	0.0029 (6)
C10D	0.0208 (8)	0.0177 (8)	0.0240 (8)	0.0025 (6)	0.0019 (6)	0.0048 (6)
C11D	0.0220 (8)	0.0178 (7)	0.0215 (8)	0.0038 (6)	0.0024 (6)	0.0058 (6)
C12D	0.0232 (9)	0.0221 (8)	0.0239 (8)	0.0025 (7)	0.0011 (7)	0.0047 (6)
C13D	0.0256 (9)	0.0314 (9)	0.0304 (9)	0.0090 (8)	0.0030 (7)	0.0105 (7)
C14D	0.0347 (10)	0.0290 (9)	0.0296 (9)	0.0165 (8)	0.0091 (8)	0.0115 (7)
C15D	0.0385 (11)	0.0201 (8)	0.0248 (8)	0.0085 (7)	0.0040 (7)	0.0048 (6)
C16C	0.0261 (9)	0.0203 (8)	0.0238 (8)	0.0051 (7)	0.0025 (7)	0.0059 (6)
C17D	0.0285 (9)	0.0268 (9)	0.0242 (8)	0.0130 (7)	−0.0023 (7)	0.0042 (7)
C18D	0.0617 (14)	0.0494 (12)	0.0197 (9)	0.0326 (11)	0.0018 (9)	0.0065 (8)
C19D	0.0778 (18)	0.0764 (18)	0.0364 (12)	0.0400 (15)	0.0188 (12)	0.0311 (12)
C20D	0.0376 (10)	0.0209 (8)	0.0279 (9)	0.0083 (7)	0.0021 (7)	0.0086 (7)
C16D	0.0272 (9)	0.0196 (8)	0.0224 (8)	0.0029 (7)	0.0003 (7)	0.0042 (6)

### Geometric parameters (Å, °)

**Table:**

S1A—C10A	1.6844 (16)	C19B—H19D	0.9600
O2A—C17A	1.210 (2)	C19B—H19E	0.9600
F2A—C14A	1.358 (2)	C19B—H19F	0.9600
N1A—C10A	1.383 (2)	C20B—H20D	0.9600
N1A—C7A	1.4219 (19)	C20B—H20E	0.9600
N1A—C6A	1.4483 (19)	C20B—H20F	0.9600
N2A—C10A	1.329 (2)	S1C—C10C	1.6786 (16)
N2A—C9A	1.467 (2)	O1C—C17C	1.339 (2)
N2A—H1NA	0.86 (2)	O1C—C18C	1.458 (2)
C1A—C6A	1.380 (2)	O2C—C17C	1.210 (2)
C1A—C2A	1.390 (3)	F1C—C12C	1.3607 (19)
C1A—H1AA	0.9300	F2C—C14C	1.3585 (19)
C2A—C3A	1.377 (3)	N1C—C10C	1.381 (2)
C2A—H2AA	0.9300	N1C—C7C	1.419 (2)
C3A—C4A	1.386 (3)	N1C—C6C	1.450 (2)
C3A—H3AA	0.9300	N2C—C10C	1.334 (2)
C4A—C5A	1.392 (2)	N2C—C9C	1.462 (2)
C4A—H4AA	0.9300	N2C—H1NC	0.90 (2)
C5A—C6A	1.382 (2)	C1C—C6C	1.380 (3)
C5A—H5AA	0.9300	C1C—C2C	1.392 (3)
C7A—C8A	1.350 (2)	C1C—H1CA	0.9300
C7A—C20A	1.507 (2)	C2C—C3C	1.361 (4)
C8A—C17A	1.481 (2)	C2C—H2CA	0.9300
C8A—C9A	1.509 (2)	C3C—C4C	1.373 (4)
C9A—C11A	1.520 (2)	C3C—H3CA	0.9300
C9A—H9AA	0.9800	C4C—C5C	1.396 (3)
C11A—C12A	1.388 (2)	C4C—H4CA	0.9300
C11A—C16A	1.390 (2)	C5C—C6C	1.374 (3)
C12A—F1A	1.325 (3)	C5C—H5CA	0.9300
C12A—C13A	1.385 (3)	C7C—C8C	1.347 (2)
C12A—H12A	0.9300	C7C—C20C	1.506 (2)
C13A—C14A	1.372 (3)	C8C—C17C	1.478 (2)
C13A—H13A	0.9300	C8C—C9C	1.513 (2)
C14A—C15A	1.367 (3)	C9C—C11C	1.520 (2)
C15A—C16A	1.385 (3)	C9C—H9CA	0.9800
C15A—H15A	0.9300	C11C—C12C	1.388 (2)
C16A—F3A	1.310 (2)	C11C—C16C	1.391 (2)
C16A—H16C	0.9300	C12C—C13C	1.382 (2)
C17A—O1A	1.327 (2)	C13C—C14C	1.381 (3)
C17A—O1E	1.523 (15)	C13C—H13C	0.9300
O1A—C18A	1.462 (2)	C14C—C15C	1.370 (3)
C18A—C19A	1.503 (3)	C15C—C16C	1.394 (2)
C18A—H18A	0.9700	C15C—H15C	0.9300
C18A—H18B	0.9700	C18C—C19C	1.504 (3)
C19A—H19A	0.9600	C18C—H18E	0.9700
C19A—H19B	0.9600	C18C—H18F	0.9700
C19A—H19C	0.9600	C19C—H19G	0.9600
O1E—C18E	1.460 (15)	C19C—H19H	0.9600
C18E—C19E	1.474 (17)	C19C—H19I	0.9600
C18E—H18I	0.9700	C20C—H20G	0.9600
C18E—H18J	0.9700	C20C—H20H	0.9600
C19E—H19M	0.9600	C20C—H20I	0.9600
C19E—H19N	0.9600	S1D—C10D	1.6777 (16)
C19E—H19O	0.9600	O1D—C17D	1.339 (2)
C20A—H20A	0.9600	O1D—C18D	1.454 (2)
C20A—H20B	0.9600	O2D—C17D	1.207 (2)
C20A—H20C	0.9600	F2D—C14D	1.3575 (19)
S1B—C10B	1.6834 (16)	F1D—C12D	1.3639 (19)
O1B—C17B	1.3350 (19)	N1D—C10D	1.3824 (19)
O1B—C18B	1.4639 (19)	N1D—C7D	1.419 (2)
O2B—C17B	1.2123 (19)	N1D—C6D	1.451 (2)
F2B—C14B	1.357 (2)	N2D—C10D	1.332 (2)
N1B—C10B	1.384 (2)	N2D—C9D	1.462 (2)
N1B—C7B	1.4237 (19)	N2D—H1ND	0.87 (2)
N1B—C6B	1.4481 (19)	C1D—C6D	1.377 (2)
N2B—C10B	1.329 (2)	C1D—C2D	1.395 (3)
N2B—C9B	1.467 (2)	C1D—H1DA	0.9300
N2B—H1NB	0.87 (2)	C2D—C3D	1.373 (3)
C1B—C6B	1.382 (2)	C2D—H2DA	0.9300
C1B—C2B	1.389 (2)	C3D—C4D	1.377 (3)
C1B—H1BA	0.9300	C3D—H3DA	0.9300
C2B—C3B	1.391 (3)	C4D—C5D	1.392 (3)
C2B—H2BA	0.9300	C4D—H4DA	0.9300
C3B—C4B	1.381 (3)	C5D—C6D	1.375 (3)
C3B—H3BA	0.9300	C5D—H5DA	0.9300
C4B—C5B	1.384 (2)	C7D—C8D	1.349 (2)
C4B—H4BA	0.9300	C7D—C20D	1.502 (2)
C5B—C6B	1.384 (2)	C8D—C17D	1.482 (2)
C5B—H5BA	0.9300	C8D—C9D	1.509 (2)
C7B—C8B	1.349 (2)	C9D—C11D	1.523 (2)
C7B—C20B	1.508 (2)	C9D—H9DA	0.9800
C8B—C17B	1.482 (2)	C11D—C12D	1.388 (2)
C8B—C9B	1.511 (2)	C11D—C16D	1.394 (2)
C9B—C11B	1.519 (2)	C12D—C13D	1.376 (2)
C9B—H9BA	0.9800	C13D—C14D	1.380 (3)
C11B—C12B	1.383 (3)	C13D—H13D	0.9300
C11B—C16B	1.389 (2)	C14D—C15D	1.374 (3)
C12B—F1B	1.311 (3)	C15D—C16D	1.393 (2)
C12B—C13B	1.385 (3)	C15D—H15D	0.9300
C12B—H12B	0.9300	C16C—H16A	0.9300
C13B—C14B	1.372 (3)	C18D—C19D	1.497 (3)
C13B—H13B	0.9300	C18D—H18G	0.9700
C14B—C15B	1.362 (3)	C18D—H18H	0.9700
C15B—C16B	1.387 (3)	C19D—H19J	0.9600
C15B—H15B	0.9300	C19D—H19K	0.9600
C16B—F3B	1.296 (3)	C19D—H19L	0.9600
C16B—H16D	0.9300	C20D—H20J	0.9600
C18B—C19B	1.500 (2)	C20D—H20K	0.9600
C18B—H18C	0.9700	C20D—H20L	0.9600
C18B—H18D	0.9700	C16D—H16B	0.9300
			
C10A—N1A—C7A	122.11 (13)	H19D—C19B—H19F	109.5
C10A—N1A—C6A	119.68 (13)	H19E—C19B—H19F	109.5
C7A—N1A—C6A	118.18 (12)	C7B—C20B—H20D	109.5
C10A—N2A—C9A	126.17 (14)	C7B—C20B—H20E	109.5
C10A—N2A—H1NA	115.5 (15)	H20D—C20B—H20E	109.5
C9A—N2A—H1NA	117.8 (15)	C7B—C20B—H20F	109.5
C6A—C1A—C2A	119.57 (18)	H20D—C20B—H20F	109.5
C6A—C1A—H1AA	120.2	H20E—C20B—H20F	109.5
C2A—C1A—H1AA	120.2	C17C—O1C—C18C	116.06 (15)
C3A—C2A—C1A	120.26 (17)	C10C—N1C—C7C	121.77 (13)
C3A—C2A—H2AA	119.9	C10C—N1C—C6C	119.11 (13)
C1A—C2A—H2AA	119.9	C7C—N1C—C6C	118.98 (13)
C2A—C3A—C4A	119.99 (17)	C10C—N2C—C9C	126.50 (14)
C2A—C3A—H3AA	120.0	C10C—N2C—H1NC	117.6 (13)
C4A—C3A—H3AA	120.0	C9C—N2C—H1NC	113.5 (13)
C3A—C4A—C5A	120.04 (18)	C6C—C1C—C2C	119.1 (2)
C3A—C4A—H4AA	120.0	C6C—C1C—H1CA	120.4
C5A—C4A—H4AA	120.0	C2C—C1C—H1CA	120.4
C6A—C5A—C4A	119.42 (16)	C3C—C2C—C1C	120.9 (2)
C6A—C5A—H5AA	120.3	C3C—C2C—H2CA	119.5
C4A—C5A—H5AA	120.3	C1C—C2C—H2CA	119.5
C1A—C6A—C5A	120.71 (15)	C2C—C3C—C4C	119.6 (2)
C1A—C6A—N1A	119.92 (15)	C2C—C3C—H3CA	120.2
C5A—C6A—N1A	119.00 (14)	C4C—C3C—H3CA	120.2
C8A—C7A—N1A	119.05 (14)	C3C—C4C—C5C	120.6 (3)
C8A—C7A—C20A	125.95 (14)	C3C—C4C—H4CA	119.7
N1A—C7A—C20A	115.00 (13)	C5C—C4C—H4CA	119.7
C7A—C8A—C17A	127.13 (14)	C6C—C5C—C4C	119.1 (2)
C7A—C8A—C9A	121.28 (14)	C6C—C5C—H5CA	120.4
C17A—C8A—C9A	111.50 (13)	C4C—C5C—H5CA	120.4
N2A—C9A—C8A	109.21 (13)	C5C—C6C—C1C	120.58 (18)
N2A—C9A—C11A	111.38 (13)	C5C—C6C—N1C	118.54 (18)
C8A—C9A—C11A	112.98 (14)	C1C—C6C—N1C	120.80 (16)
N2A—C9A—H9AA	107.7	C8C—C7C—N1C	119.93 (14)
C8A—C9A—H9AA	107.7	C8C—C7C—C20C	125.62 (15)
C11A—C9A—H9AA	107.7	N1C—C7C—C20C	114.36 (14)
N2A—C10A—N1A	116.49 (14)	C7C—C8C—C17C	125.96 (14)
N2A—C10A—S1A	121.62 (12)	C7C—C8C—C9C	121.75 (14)
N1A—C10A—S1A	121.87 (12)	C17C—C8C—C9C	112.28 (14)
C12A—C11A—C16A	116.12 (16)	N2C—C9C—C8C	109.13 (13)
C12A—C11A—C9A	122.20 (14)	N2C—C9C—C11C	111.92 (12)
C16A—C11A—C9A	121.68 (16)	C8C—C9C—C11C	113.61 (13)
F1A—C12A—C13A	117.73 (19)	N2C—C9C—H9CA	107.3
F1A—C12A—C11A	117.79 (19)	C8C—C9C—H9CA	107.3
C13A—C12A—C11A	123.27 (16)	C11C—C9C—H9CA	107.3
C13A—C12A—H12A	118.4	N2C—C10C—N1C	116.46 (14)
C11A—C12A—H12A	118.4	N2C—C10C—S1C	121.64 (12)
C14A—C13A—C12A	117.16 (18)	N1C—C10C—S1C	121.79 (12)
C14A—C13A—H13A	121.4	C12C—C11C—C16C	116.66 (15)
C12A—C13A—H13A	121.4	C12C—C11C—C9C	122.63 (14)
F2A—C14A—C15A	118.70 (16)	C16C—C11C—C9C	120.71 (15)
F2A—C14A—C13A	118.34 (18)	F1C—C12C—C13C	117.59 (15)
C15A—C14A—C13A	122.95 (17)	F1C—C12C—C11C	118.72 (14)
C14A—C15A—C16A	117.85 (16)	C13C—C12C—C11C	123.68 (16)
C14A—C15A—H15A	121.1	C14C—C13C—C12C	116.63 (16)
C16A—C15A—H15A	121.1	C14C—C13C—H13C	121.7
F3A—C16A—C15A	117.58 (17)	C12C—C13C—H13C	121.7
F3A—C16A—C11A	119.60 (18)	F2C—C14C—C15C	119.22 (16)
C15A—C16A—C11A	122.65 (18)	F2C—C14C—C13C	117.68 (16)
C15A—C16A—H16C	118.7	C15C—C14C—C13C	123.10 (16)
C11A—C16A—H16C	118.7	C14C—C15C—C16C	117.98 (16)
O2A—C17A—O1A	122.39 (15)	C14C—C15C—H15C	121.0
O2A—C17A—C8A	121.07 (15)	C16C—C15C—H15C	121.0
O1A—C17A—C8A	116.46 (14)	O2C—C17C—O1C	123.25 (16)
O2A—C17A—O1E	126.1 (5)	O2C—C17C—C8C	122.56 (16)
C8A—C17A—O1E	105.1 (5)	O1C—C17C—C8C	114.09 (15)
C17A—O1A—C18A	115.92 (14)	O1C—C18C—C19C	106.16 (17)
O1A—C18A—C19A	106.50 (15)	O1C—C18C—H18E	110.5
O1A—C18A—H18A	110.4	C19C—C18C—H18E	110.5
C19A—C18A—H18A	110.4	O1C—C18C—H18F	110.5
O1A—C18A—H18B	110.4	C19C—C18C—H18F	110.5
C19A—C18A—H18B	110.4	H18E—C18C—H18F	108.7
H18A—C18A—H18B	108.6	C18C—C19C—H19G	109.5
C18E—O1E—C17A	113.7 (13)	C18C—C19C—H19H	109.5
C19E—C18E—O1E	108.7 (14)	H19G—C19C—H19H	109.5
C19E—C18E—H18I	110.0	C18C—C19C—H19I	109.5
O1E—C18E—H18I	110.0	H19G—C19C—H19I	109.5
C19E—C18E—H18J	110.0	H19H—C19C—H19I	109.5
O1E—C18E—H18J	110.0	C7C—C20C—H20G	109.5
H18I—C18E—H18J	108.3	C7C—C20C—H20H	109.5
C18E—C19E—H19M	109.5	H20G—C20C—H20H	109.5
C18E—C19E—H19N	109.5	C7C—C20C—H20I	109.5
H19M—C19E—H19N	109.5	H20G—C20C—H20I	109.5
C18E—C19E—H19O	109.5	H20H—C20C—H20I	109.5
H19M—C19E—H19O	109.5	C17D—O1D—C18D	116.44 (15)
H19N—C19E—H19O	109.5	C10D—N1D—C7D	122.12 (13)
C7A—C20A—H20A	109.5	C10D—N1D—C6D	117.90 (13)
C7A—C20A—H20B	109.5	C7D—N1D—C6D	119.59 (13)
H20A—C20A—H20B	109.5	C10D—N2D—C9D	126.23 (14)
C7A—C20A—H20C	109.5	C10D—N2D—H1ND	119.4 (13)
H20A—C20A—H20C	109.5	C9D—N2D—H1ND	111.7 (13)
H20B—C20A—H20C	109.5	C6D—C1D—C2D	119.13 (19)
C17B—O1B—C18B	115.51 (13)	C6D—C1D—H1DA	120.4
C10B—N1B—C7B	122.06 (13)	C2D—C1D—H1DA	120.4
C10B—N1B—C6B	118.97 (13)	C3D—C2D—C1D	120.47 (19)
C7B—N1B—C6B	118.95 (13)	C3D—C2D—H2DA	119.8
C10B—N2B—C9B	126.02 (14)	C1D—C2D—H2DA	119.8
C10B—N2B—H1NB	117.6 (13)	C2D—C3D—C4D	119.87 (19)
C9B—N2B—H1NB	115.0 (13)	C2D—C3D—H3DA	120.1
C6B—C1B—C2B	119.55 (15)	C4D—C3D—H3DA	120.1
C6B—C1B—H1BA	120.2	C3D—C4D—C5D	120.2 (2)
C2B—C1B—H1BA	120.2	C3D—C4D—H4DA	119.9
C1B—C2B—C3B	119.92 (17)	C5D—C4D—H4DA	119.9
C1B—C2B—H2BA	120.0	C6D—C5D—C4D	119.50 (19)
C3B—C2B—H2BA	120.0	C6D—C5D—H5DA	120.3
C4B—C3B—C2B	119.86 (16)	C4D—C5D—H5DA	120.3
C4B—C3B—H3BA	120.1	C5D—C6D—C1D	120.83 (17)
C2B—C3B—H3BA	120.1	C5D—C6D—N1D	117.91 (15)
C3B—C4B—C5B	120.47 (16)	C1D—C6D—N1D	121.24 (16)
C3B—C4B—H4BA	119.8	C8D—C7D—N1D	119.46 (14)
C5B—C4B—H4BA	119.8	C8D—C7D—C20D	126.84 (15)
C6B—C5B—C4B	119.44 (16)	N1D—C7D—C20D	113.58 (14)
C6B—C5B—H5BA	120.3	C7D—C8D—C17D	126.09 (15)
C4B—C5B—H5BA	120.3	C7D—C8D—C9D	121.85 (14)
C1B—C6B—C5B	120.76 (15)	C17D—C8D—C9D	112.04 (14)
C1B—C6B—N1B	119.10 (14)	N2D—C9D—C8D	109.30 (13)
C5B—C6B—N1B	119.97 (15)	N2D—C9D—C11D	112.04 (12)
C8B—C7B—N1B	119.36 (14)	C8D—C9D—C11D	113.85 (13)
C8B—C7B—C20B	125.95 (14)	N2D—C9D—H9DA	107.1
N1B—C7B—C20B	114.69 (13)	C8D—C9D—H9DA	107.1
C7B—C8B—C17B	127.42 (15)	C11D—C9D—H9DA	107.1
C7B—C8B—C9B	121.09 (14)	N2D—C10D—N1D	116.46 (14)
C17B—C8B—C9B	111.44 (13)	N2D—C10D—S1D	122.06 (12)
N2B—C9B—C8B	109.39 (13)	N1D—C10D—S1D	121.34 (12)
N2B—C9B—C11B	110.81 (13)	C12D—C11D—C16D	116.66 (15)
C8B—C9B—C11B	113.25 (14)	C12D—C11D—C9D	122.91 (14)
N2B—C9B—H9BA	107.7	C16D—C11D—C9D	120.42 (14)
C8B—C9B—H9BA	107.7	F1D—C12D—C13D	117.75 (15)
C11B—C9B—H9BA	107.7	F1D—C12D—C11D	118.55 (14)
N2B—C10B—N1B	116.59 (14)	C13D—C12D—C11D	123.69 (16)
N2B—C10B—S1B	121.74 (12)	C12D—C13D—C14D	116.90 (17)
N1B—C10B—S1B	121.64 (12)	C12D—C13D—H13D	121.6
C12B—C11B—C16B	116.02 (17)	C14D—C13D—H13D	121.6
C12B—C11B—C9B	122.21 (15)	F2D—C14D—C15D	119.14 (16)
C16B—C11B—C9B	121.76 (16)	F2D—C14D—C13D	117.89 (17)
F1B—C12B—C11B	116.75 (19)	C15D—C14D—C13D	122.97 (16)
F1B—C12B—C13B	118.0 (2)	C14D—C15D—C16D	117.94 (16)
C11B—C12B—C13B	123.32 (18)	C14D—C15D—H15D	121.0
C11B—C12B—H12B	118.3	C16D—C15D—H15D	121.0
C13B—C12B—H12B	118.3	C11C—C16C—C15C	121.89 (16)
C14B—C13B—C12B	117.3 (2)	C11C—C16C—H16A	119.1
C14B—C13B—H13B	121.3	C15C—C16C—H16A	119.1
C12B—C13B—H13B	121.3	O2D—C17D—O1D	123.09 (16)
F2B—C14B—C15B	118.68 (18)	O2D—C17D—C8D	122.62 (16)
F2B—C14B—C13B	118.6 (2)	O1D—C17D—C8D	114.16 (15)
C15B—C14B—C13B	122.72 (18)	O1D—C18D—C19D	105.99 (18)
C14B—C15B—C16B	117.95 (18)	O1D—C18D—H18G	110.5
C14B—C15B—H15B	121.0	C19D—C18D—H18G	110.5
C16B—C15B—H15B	121.0	O1D—C18D—H18H	110.5
F3B—C16B—C15B	117.85 (18)	C19D—C18D—H18H	110.5
F3B—C16B—C11B	119.27 (19)	H18G—C18D—H18H	108.7
C15B—C16B—C11B	122.7 (2)	C18D—C19D—H19J	109.5
C15B—C16B—H16D	118.7	C18D—C19D—H19K	109.5
C11B—C16B—H16D	118.7	H19J—C19D—H19K	109.5
O2B—C17B—O1B	122.80 (15)	C18D—C19D—H19L	109.5
O2B—C17B—C8B	120.63 (15)	H19J—C19D—H19L	109.5
O1B—C17B—C8B	116.57 (14)	H19K—C19D—H19L	109.5
O1B—C18B—C19B	107.26 (14)	C7D—C20D—H20J	109.5
O1B—C18B—H18C	110.3	C7D—C20D—H20K	109.5
C19B—C18B—H18C	110.3	H20J—C20D—H20K	109.5
O1B—C18B—H18D	110.3	C7D—C20D—H20L	109.5
C19B—C18B—H18D	110.3	H20J—C20D—H20L	109.5
H18C—C18B—H18D	108.5	H20K—C20D—H20L	109.5
C18B—C19B—H19D	109.5	C15D—C16D—C11D	121.80 (16)
C18B—C19B—H19E	109.5	C15D—C16D—H16B	119.1
H19D—C19B—H19E	109.5	C11D—C16D—H16B	119.1
C18B—C19B—H19F	109.5		
			
C6A—C1A—C2A—C3A	0.8 (3)	C18B—O1B—C17B—C8B	177.07 (14)
C1A—C2A—C3A—C4A	−0.2 (3)	C7B—C8B—C17B—O2B	170.72 (17)
C2A—C3A—C4A—C5A	0.0 (3)	C9B—C8B—C17B—O2B	−6.5 (2)
C3A—C4A—C5A—C6A	−0.4 (3)	C7B—C8B—C17B—O1B	−9.4 (3)
C2A—C1A—C6A—C5A	−1.2 (3)	C9B—C8B—C17B—O1B	173.40 (14)
C2A—C1A—C6A—N1A	171.70 (16)	C17B—O1B—C18B—C19B	178.89 (15)
C4A—C5A—C6A—C1A	1.0 (2)	C6C—C1C—C2C—C3C	−2.2 (3)
C4A—C5A—C6A—N1A	−171.93 (15)	C1C—C2C—C3C—C4C	1.0 (3)
C10A—N1A—C6A—C1A	87.56 (19)	C2C—C3C—C4C—C5C	1.2 (4)
C7A—N1A—C6A—C1A	−90.26 (19)	C3C—C4C—C5C—C6C	−2.2 (4)
C10A—N1A—C6A—C5A	−99.42 (18)	C4C—C5C—C6C—C1C	1.0 (3)
C7A—N1A—C6A—C5A	82.75 (19)	C4C—C5C—C6C—N1C	−175.91 (19)
C10A—N1A—C7A—C8A	14.0 (2)	C2C—C1C—C6C—C5C	1.1 (3)
C6A—N1A—C7A—C8A	−168.28 (15)	C2C—C1C—C6C—N1C	177.95 (15)
C10A—N1A—C7A—C20A	−165.36 (15)	C10C—N1C—C6C—C5C	−107.12 (19)
C6A—N1A—C7A—C20A	12.4 (2)	C7C—N1C—C6C—C5C	68.7 (2)
N1A—C7A—C8A—C17A	178.96 (15)	C10C—N1C—C6C—C1C	76.0 (2)
C20A—C7A—C8A—C17A	−1.8 (3)	C7C—N1C—C6C—C1C	−108.22 (18)
N1A—C7A—C8A—C9A	2.6 (2)	C10C—N1C—C7C—C8C	9.8 (2)
C20A—C7A—C8A—C9A	−178.18 (16)	C6C—N1C—C7C—C8C	−165.86 (15)
C10A—N2A—C9A—C8A	26.3 (2)	C10C—N1C—C7C—C20C	−166.89 (15)
C10A—N2A—C9A—C11A	−99.17 (18)	C6C—N1C—C7C—C20C	17.4 (2)
C7A—C8A—C9A—N2A	−20.1 (2)	N1C—C7C—C8C—C17C	178.20 (15)
C17A—C8A—C9A—N2A	163.02 (14)	C20C—C7C—C8C—C17C	−5.5 (3)
C7A—C8A—C9A—C11A	104.46 (18)	N1C—C7C—C8C—C9C	−3.3 (2)
C17A—C8A—C9A—C11A	−72.43 (17)	C20C—C7C—C8C—C9C	173.04 (15)
C9A—N2A—C10A—N1A	−12.7 (2)	C10C—N2C—C9C—C8C	25.2 (2)
C9A—N2A—C10A—S1A	168.72 (13)	C10C—N2C—C9C—C11C	−101.43 (17)
C7A—N1A—C10A—N2A	−9.3 (2)	C7C—C8C—C9C—N2C	−12.0 (2)
C6A—N1A—C10A—N2A	172.99 (14)	C17C—C8C—C9C—N2C	166.75 (13)
C7A—N1A—C10A—S1A	169.28 (12)	C7C—C8C—C9C—C11C	113.68 (17)
C6A—N1A—C10A—S1A	−8.5 (2)	C17C—C8C—C9C—C11C	−67.59 (18)
N2A—C9A—C11A—C12A	73.41 (19)	C9C—N2C—C10C—N1C	−20.7 (2)
C8A—C9A—C11A—C12A	−49.9 (2)	C9C—N2C—C10C—S1C	163.05 (12)
N2A—C9A—C11A—C16A	−106.84 (17)	C7C—N1C—C10C—N2C	1.4 (2)
C8A—C9A—C11A—C16A	129.81 (16)	C6C—N1C—C10C—N2C	177.09 (14)
C16A—C11A—C12A—F1A	166.80 (19)	C7C—N1C—C10C—S1C	177.69 (12)
C9A—C11A—C12A—F1A	−13.4 (3)	C6C—N1C—C10C—S1C	−6.6 (2)
C16A—C11A—C12A—C13A	−0.3 (3)	N2C—C9C—C11C—C12C	54.2 (2)
C9A—C11A—C12A—C13A	179.47 (16)	C8C—C9C—C11C—C12C	−70.00 (19)
F1A—C12A—C13A—C14A	−166.5 (2)	N2C—C9C—C11C—C16C	−125.58 (16)
C11A—C12A—C13A—C14A	0.6 (3)	C8C—C9C—C11C—C16C	110.26 (17)
C12A—C13A—C14A—F2A	179.39 (16)	C16C—C11C—C12C—F1C	−179.65 (14)
C12A—C13A—C14A—C15A	−0.3 (3)	C9C—C11C—C12C—F1C	0.6 (2)
F2A—C14A—C15A—C16A	−179.91 (15)	C16C—C11C—C12C—C13C	0.6 (2)
C13A—C14A—C15A—C16A	−0.2 (3)	C9C—C11C—C12C—C13C	−179.18 (15)
C14A—C15A—C16A—F3A	175.75 (17)	F1C—C12C—C13C—C14C	177.93 (14)
C14A—C15A—C16A—C11A	0.5 (3)	C11C—C12C—C13C—C14C	−2.3 (3)
C12A—C11A—C16A—F3A	−175.39 (17)	C12C—C13C—C14C—F2C	−177.77 (14)
C9A—C11A—C16A—F3A	4.8 (2)	C12C—C13C—C14C—C15C	2.3 (3)
C12A—C11A—C16A—C15A	−0.3 (2)	F2C—C14C—C15C—C16C	179.43 (14)
C9A—C11A—C16A—C15A	179.97 (15)	C13C—C14C—C15C—C16C	−0.7 (3)
C7A—C8A—C17A—O2A	−173.23 (17)	C18C—O1C—C17C—O2C	−2.7 (2)
C9A—C8A—C17A—O2A	3.4 (2)	C18C—O1C—C17C—C8C	−179.13 (14)
C7A—C8A—C17A—O1A	3.7 (3)	C7C—C8C—C17C—O2C	158.63 (17)
C9A—C8A—C17A—O1A	−179.66 (16)	C9C—C8C—C17C—O2C	−20.0 (2)
C7A—C8A—C17A—O1E	35.7 (7)	C7C—C8C—C17C—O1C	−24.9 (2)
C9A—C8A—C17A—O1E	−147.7 (7)	C9C—C8C—C17C—O1C	156.40 (14)
O2A—C17A—O1A—C18A	0.4 (3)	C17C—O1C—C18C—C19C	−168.78 (15)
C8A—C17A—O1A—C18A	−176.45 (16)	C6D—C1D—C2D—C3D	−0.4 (3)
O1E—C17A—O1A—C18A	108.0 (9)	C1D—C2D—C3D—C4D	−1.7 (3)
C17A—O1A—C18A—C19A	−176.8 (2)	C2D—C3D—C4D—C5D	2.6 (3)
O2A—C17A—O1E—C18E	34.5 (16)	C3D—C4D—C5D—C6D	−1.4 (3)
O1A—C17A—O1E—C18E	−60.2 (12)	C4D—C5D—C6D—C1D	−0.7 (3)
C8A—C17A—O1E—C18E	−176.3 (11)	C4D—C5D—C6D—N1D	−179.23 (16)
C17A—O1E—C18E—C19E	85 (2)	C2D—C1D—C6D—C5D	1.5 (3)
C6B—C1B—C2B—C3B	−0.1 (2)	C2D—C1D—C6D—N1D	−179.93 (15)
C1B—C2B—C3B—C4B	0.0 (3)	C10D—N1D—C6D—C5D	90.06 (19)
C2B—C3B—C4B—C5B	0.3 (3)	C7D—N1D—C6D—C5D	−82.95 (19)
C3B—C4B—C5B—C6B	−0.5 (3)	C10D—N1D—C6D—C1D	−88.51 (19)
C2B—C1B—C6B—C5B	−0.1 (2)	C7D—N1D—C6D—C1D	98.48 (19)
C2B—C1B—C6B—N1B	175.12 (14)	C10D—N1D—C7D—C8D	−9.7 (2)
C4B—C5B—C6B—C1B	0.4 (2)	C6D—N1D—C7D—C8D	162.99 (15)
C4B—C5B—C6B—N1B	−174.76 (15)	C10D—N1D—C7D—C20D	166.60 (15)
C10B—N1B—C6B—C1B	92.31 (18)	C6D—N1D—C7D—C20D	−20.7 (2)
C7B—N1B—C6B—C1B	−88.92 (18)	N1D—C7D—C8D—C17D	179.80 (15)
C10B—N1B—C6B—C5B	−92.45 (18)	C20D—C7D—C8D—C17D	4.0 (3)
C7B—N1B—C6B—C5B	86.31 (19)	N1D—C7D—C8D—C9D	1.9 (2)
C10B—N1B—C7B—C8B	−12.3 (2)	C20D—C7D—C8D—C9D	−173.87 (16)
C6B—N1B—C7B—C8B	168.95 (15)	C10D—N2D—C9D—C8D	−25.7 (2)
C10B—N1B—C7B—C20B	167.73 (15)	C10D—N2D—C9D—C11D	101.43 (18)
C6B—N1B—C7B—C20B	−11.0 (2)	C7D—C8D—C9D—N2D	13.5 (2)
N1B—C7B—C8B—C17B	−179.67 (15)	C17D—C8D—C9D—N2D	−164.67 (13)
C20B—C7B—C8B—C17B	0.3 (3)	C7D—C8D—C9D—C11D	−112.62 (18)
N1B—C7B—C8B—C9B	−2.7 (2)	C17D—C8D—C9D—C11D	69.20 (18)
C20B—C7B—C8B—C9B	177.27 (15)	C9D—N2D—C10D—N1D	20.1 (2)
C10B—N2B—C9B—C8B	−26.8 (2)	C9D—N2D—C10D—S1D	−164.18 (12)
C10B—N2B—C9B—C11B	98.75 (19)	C7D—N1D—C10D—N2D	−0.6 (2)
C7B—C8B—C9B—N2B	19.7 (2)	C6D—N1D—C10D—N2D	−173.40 (14)
C17B—C8B—C9B—N2B	−162.91 (13)	C7D—N1D—C10D—S1D	−176.36 (12)
C7B—C8B—C9B—C11B	−104.48 (18)	C6D—N1D—C10D—S1D	10.8 (2)
C17B—C8B—C9B—C11B	72.96 (17)	N2D—C9D—C11D—C12D	−53.4 (2)
C9B—N2B—C10B—N1B	14.6 (2)	C8D—C9D—C11D—C12D	71.2 (2)
C9B—N2B—C10B—S1B	−167.28 (13)	N2D—C9D—C11D—C16D	125.69 (15)
C7B—N1B—C10B—N2B	6.8 (2)	C8D—C9D—C11D—C16D	−109.64 (17)
C6B—N1B—C10B—N2B	−174.44 (14)	C16D—C11D—C12D—F1D	179.49 (14)
C7B—N1B—C10B—S1B	−171.33 (12)	C9D—C11D—C12D—F1D	−1.4 (2)
C6B—N1B—C10B—S1B	7.4 (2)	C16D—C11D—C12D—C13D	−1.5 (2)
N2B—C9B—C11B—C12B	−74.1 (2)	C9D—C11D—C12D—C13D	177.69 (15)
C8B—C9B—C11B—C12B	49.3 (2)	F1D—C12D—C13D—C14D	−178.42 (15)
N2B—C9B—C11B—C16B	105.33 (17)	C11D—C12D—C13D—C14D	2.5 (3)
C8B—C9B—C11B—C16B	−131.32 (16)	C12D—C13D—C14D—F2D	177.85 (15)
C16B—C11B—C12B—F1B	−163.7 (2)	C12D—C13D—C14D—C15D	−1.8 (3)
C9B—C11B—C12B—F1B	15.8 (3)	F2D—C14D—C15D—C16D	−179.53 (15)
C16B—C11B—C12B—C13B	0.4 (3)	C13D—C14D—C15D—C16D	0.1 (3)
C9B—C11B—C12B—C13B	179.81 (17)	C12C—C11C—C16C—C15C	1.2 (2)
F1B—C12B—C13B—C14B	163.0 (2)	C9C—C11C—C16C—C15C	−179.01 (14)
C11B—C12B—C13B—C14B	−0.8 (3)	C14C—C15C—C16C—C11C	−1.2 (2)
C12B—C13B—C14B—F2B	−178.93 (17)	C18D—O1D—C17D—O2D	4.0 (2)
C12B—C13B—C14B—C15B	0.6 (3)	C18D—O1D—C17D—C8D	−179.82 (14)
F2B—C14B—C15B—C16B	179.56 (16)	C7D—C8D—C17D—O2D	−157.11 (18)
C13B—C14B—C15B—C16B	0.0 (3)	C9D—C8D—C17D—O2D	21.0 (2)
C14B—C15B—C16B—F3B	−175.16 (18)	C7D—C8D—C17D—O1D	26.7 (2)
C14B—C15B—C16B—C11B	−0.5 (3)	C9D—C8D—C17D—O1D	−155.20 (14)
C12B—C11B—C16B—F3B	174.88 (18)	C17D—O1D—C18D—C19D	167.69 (16)
C9B—C11B—C16B—F3B	−4.6 (3)	C14D—C15D—C16D—C11D	1.0 (2)
C12B—C11B—C16B—C15B	0.3 (3)	C12D—C11D—C16D—C15D	−0.4 (2)
C9B—C11B—C16B—C15B	−179.13 (16)	C9D—C11D—C16D—C15D	−179.55 (15)
C18B—O1B—C17B—O2B	−3.0 (2)		

### Hydrogen-bond geometry (Å, °)

**Table:**

*D*—H···*A*	*D*—H	H···*A*	*D*···*A*	*D*—H···*A*
N2A—H1NA···S1B	0.86 (2)	2.63 (2)	3.4521 (15)	161.1 (19)
N2B—H1NB···S1A	0.87 (2)	2.624 (19)	3.4632 (15)	163.1 (18)
N2C—H1NC···S1C^i^	0.90 (2)	2.41 (2)	3.2686 (15)	160.0 (17)
N2D—H1ND···S1D^ii^	0.87 (2)	2.42 (2)	3.2537 (15)	159.7 (17)
C9B—H9BA···O2C^iii^	0.98	2.50	3.199 (2)	128
C16C—H16A···O2A^i^	0.93	2.46	3.143 (2)	130
C16D—H16B···O2B^iv^	0.93	2.47	3.146 (2)	130
C18A—H18B···O2B^v^	0.97	2.52	3.446 (3)	159
C18B—H18C···O2A^vi^	0.97	2.50	3.427 (2)	160

Symmetry codes: (i) −*x*+2, −*y*+2, −*z*+1; (ii) −*x*+1, −*y*+1, −*z*+2; (iii) −*x*+2, −*y*+1, −*z*+1; (iv) −*x*+1, −*y*, −*z*+1; (v) *x*, *y*+1, *z*; (vi) *x*, *y*−1, *z*.

## References

[bb1] Allen, F. H., Kennard, O., Watson, D. G., Brammer, L., Orpen, A. G. & Taylor, R. (1987). *J. Chem. Soc. Perkin Trans. 2*, pp. S1–S19.

[bb2] Atwal, K. S. (1990). *J. Med. Chem.* **33**, 1510–1515. 10.1021/jm00167a035 2329573

[bb3] Bruker (2005). *APEX2*, *SAINT* and *SADABS* Bruker AXS Inc., Madison, Wisconsin, USA.

[bb4] Cosier, J. & Glazer, A. M. (1986). *J. Appl. Cryst.* **19**, 105–107.

[bb5] Cremer, D. & Pople, J. A. (1975). *J. Am. Chem. Soc.* **97**, 1354–1358.

[bb6] Kalluraya, B. & Rai, G. (2003). *Synth. Commun.* **33**, 3589–3595.

[bb7] Kappe, C. O. (1993). *Tetrahedron*, **49**, 6937–6963.

[bb8] Manjula, A., Rao, B. V. & Neelakantan, P. (2004). *Synth. Commun.* **34**, 2665–2671.

[bb9] Sadanandam, Y. S., Shetty, M. M. & Diwan, P. V. (1992). *Eur. J. Med. Chem.* **27**, 87–92.

[bb10] Sheldrick, G. M. (2008). *Acta Cryst.* A**64**, 112–122. 10.1107/S0108767307043930 18156677

[bb11] Spek, A. L. (2009). *Acta Cryst.* D**65**, 148–155. 10.1107/S090744490804362X PMC2631630 19171970

[bb12] Steele, T. G., Coburn, C. A., Patane, M. A. & Bock, M. G. (1998). *Tetrahedron Lett.* **39**, 9315–9318.

